# A Study on the Architecture of Artificial Neural Network Considering Injection-Molding Process Steps

**DOI:** 10.3390/polym15234578

**Published:** 2023-11-30

**Authors:** Junhan Lee, Jongsun Kim, Jongsu Kim

**Affiliations:** Molding & Metal Forming R & D Department, Korea Institute of Industrial Technology, Bucheon 14442, Republic of Korea; jhlee0526@kitech.re.kr (J.L.); libra74@kitech.re.kr (J.K.)

**Keywords:** quality prediction, multi-task learning, input parameter, multi-input, learning structure, optimization, injection step, packing step, cooling step

## Abstract

In this study, an artificial neural network (ANN) was established to predict product properties (mass, diameter, height) using six process conditions of the injection-molding process (melt temperature, mold temperature, injection speed, packing pressure, packing time, and cooling time) as input parameters. The injection-molding process consists of continuous sequential stages, including the injection stage, packing stage, and cooling stage. However, the related research tends to have an insufficient incorporation of structural characteristics based on these basic process stages. Therefore, in order to incorporate these process stages and characteristics into the ANN, a process-based multi-task learning technique was applied to the connection between the input parameters and the front-end of the hidden layer. This resulted in the construction of two network structures, and their performance was evaluated by comparing them with the typical network structure. The results showed that a multi-task learning architecture that incorporated process-level specific structures in the connections between the input parameters and the front end of the hidden layer yielded relatively better root mean square errors (RMSEs) values than a conventional neural network architecture, by as much as two orders of magnitude. Based on these results, this study has provided guidance for the construction of artificial neural networks for injection-molding processes that incorporates process-stage specific features and structures in the architecture.

## 1. Introduction

Injection molding is one of the most prominent processes in the plastics manufacturing industry, known for its various advantages, such as short production times, high-volume production, and high dimensional accuracy [1,2]. In this process, plastics are heated above their melting point to a molten state and then injected into cavities within a mold at high speeds and pressures. Given the characteristics of this process, variables such as melt temperature, mold temperature, and injection speed are critical factors in determining the quality of molded products. Therefore, optimizing these factors is a critical process for improving product quality and productivity in the injection-molding process [3,4,5,6].

To address this, computer-aided engineering (CAE) technology has been used to model the relationships between injection-molding variables and the final product quality and to optimize the process [7,8,9,10]. However, the analysis process of injection-molding simulations using CAE involves numerous assumptions and often requires a significant amount of computation time to accurately predict the final dimensions. In addition, due to the inherent nonlinear characteristics of plastic resin and the injection-molding process, there is a practical disparity between the predicted values for product characteristics such as warpage and dimensions and their actual results. As a result, there has always been a need for novel approaches to optimize the production of injection-molded products with specific quality targets such as mass or length. Recently, there has been a growing trend for applying artificial neural network (ANN) technology to model and optimize the relationships between input variables, such as resin temperature in injection-molding processes, and output variables, such as mass or specific lengths. ANNs are a prominent technique in the field of artificial intelligence, known for their robust performance in uncovering complex, inherently nonlinear relationships. They find widespread applications in various domains, including data mining, image processing, modeling of engineering systems, and engineering control. Research efforts are also underway to apply ANNs to injection-molding processes, focusing on optimizing the relationship between process conditions and the molded product quality.

Ozcelik et al. [11] used a multi-input, single-output (MISO) structure to construct an ANN with mold temperature, melt temperature, packing pressure, packing time, and cooling time as input parameters. They predicted process conditions to minimize warpage and verified the extent to which warpage was minimized by practical experiments. Yin et al. [12] utilized identical sets of five input parameters and derived the warpage data for the automotive glove compartment cap from CAE results, as opposed to relying on experiments. Lee et al. [13] utilized shape-related data, including volume and area, alongside the conventional six process conditions as input variables to anticipate the mass of a product across various molds. The artificial neural network (ANN) was constructed by incorporating both experimental data and computer-aided engineering (CAE) analysis results. Leveraging the ANN model, they achieved favorable outcomes in establishing a system for determining the optimal combination of input parameters applicable to molds of diverse shapes. Gim et al. [14] employed sensors to measure cavity pressure and time. Subsequently, they identified five specific points—namely, the start of the filling stage, switchover point, maximum cavity pressure, packing endpoint, and cooling endpoint. These points were chosen to extract pressure and time values, serving as input parameters for an artificial neural network (ANN) structure. The ANN was designed to predict the mass of the injection-molded product (specifically a spiral) as the output parameter, using a multi-input, single-output (MISO) approach. Furthermore, their research included a study on optimizing the molding window through sensitivity analysis, yielding positive outcomes. Table 1 lists studies in which MISO models are applied to the injection-molding process.

Recently, research on multi-input, multi-output (MIMO)-structured ANNs has been actively pursued, as seen in studies such as Abdul et al. [15] and Heinisch et al. [16], aimed at predicting multiple target qualities under different process conditions. Table 2 lists studies in which MIMO models are applied to the injection-molding process.

In most related studies, when the process conditions applied to injection-molding processes were used as input variables, the architecture was constructed by providing them to the hidden layers as a single task, without distinguishing between process stages or features. However, the injection-molding process is essentially a continuous sequence of stages, including injection, packaging, and cooling. The effect of process conditions on each of these stages is different, and the preceding stage can also affect the subsequent stage. In other words, if the input parameters of the injection, packing, and cooling stages, which have different processes and characteristics, are simply applied to the neural network as a group without distinguishing between the processes, it may be difficult to distinguish the degree to which each process step affects the quality of the product and update the weights and biases accordingly. From a microscopic point of view, each input parameter, melt temperature, injection speed, etc., affects the quality of the product, but from a macroscopic point of view, the quality of the product may vary depending on the interaction and feedback of each process step, injection stage, packing stage, etc., so it may be necessary to reflect the structure and characteristics of the process step at the macro level in the ANN architecture. For example, the level of packing pressure affects the quality of the product, but, from a macro perspective, it can also be analyzed that the quality of the product depends on how the packing pressures are applied and interact during the packing phase. However, in single-task learning with an undifferentiated input parameter structure, this may be difficult to reflect in the update of the ANN. Therefore, although detailed knowledge and experience of the injection-molding process may not be necessary to construct ANNs, it may be necessary to construct an ANN by considering the basic process steps to achieve structural optimization for the neural network.

**Figure 1:**
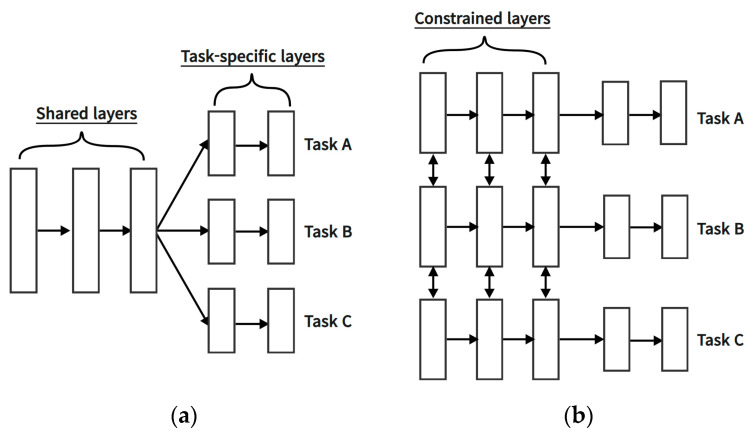
Multi-task learning architecture in deep learning: (**a**) hard-parameter sharing; (**b**) soft-parameter sharing [19,20].

To improve this, in this study, two different architectures were constructed to separately address input parameters influencing the injection stage, those affecting the packing stage, and those impacting the cooling stage in the existing artificial neural network structure. Then, the predictive performance was compared with that of the conventional neural network, and an analysis was conducted on the importance of incorporating considerations for process stages in the construction of artificial neural networks. The data-based intelligent neural networks algorithm developed to minimize bubble defects occurring in curved multi-display atmospheric bonding equipment under atmospheric environments was applied to the injection-molding process. In order to incorporate the effect of each process stage of the injection-molding process into the structure of the ANN, a multi-task learning technique was applied to the connection between the input parameters and the front of the hidden layer. The methods for constructing ANN models can be categorized into single-task learning and multi-task learning. Single-task learning involves a structure in which the input layer, hidden layer, and output layer are connected for a single task. Initially, the majority of neural network models were constructed using this single-task learning approach. However, when integrating variables with inherently different characteristics into a single task, during the update process of the ANN model, the features of all variables become interrelated, leading to changes in the weights and biases of that layer. Consequently, accurately reflecting the unique characteristics of each variable in the model becomes challenging. As an example, when weights and biases are adjusted to enhance the prediction accuracy of one output, the prediction accuracy of other outputs may decrease. To address the shortcomings of single-task learning, a proposed method is multi-task learning. In ANN models that incorporate multi-task learning, learning does not occur solely for one task. Instead, variables are organized into separate layers as needed, and multiple tasks are structured to progress independently, all within a single ANN model, as shown Figure 1. Multi-task learning allows individualized learning for each variable, distinguishing them by their characteristics and creating separate tasks. This approach can lead to the construction of more efficient and appropriate ANN models for each variable, as opposed to single-task learning. In this study, the performance of ANNs based on process-specific architectures was evaluated by applying multi-task learning at the front-end of shared layers to account for the sequence-specific features of injection-molding processes in the input parameters.

An ANN with a multi-task learning structure was constructed by categorizing the six process conditions—melt temperature, mold temperature, injection speed, packing pressure, packing time, and cooling time—into injection phase, packing phase and cooling phase. Based on this, two new structures were constructed that incorporated the process stages into the connection between the input parameters and the front-end of the hidden layer, and their performance was compared with the conventional neural network structure. The output parameters included the mass, diameter, and height of the molded product. In order to compare the performance in different scenarios, a MISO neural network with single output parameters for mass, diameter, and height were constructed and their performance was compared. In addition, based on this, the need and guidance for integrating process stage-specific structures into ANN architectures for injection-molding processes were provided.

## 2. Experiment

### 2.1. Material and Molding Equipment

**Figure 2:**
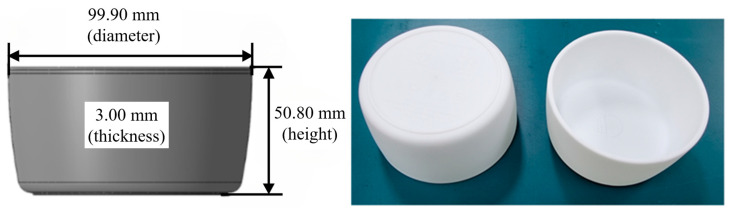
Images of the bowl used in this study.

In this study, injection-molding experiments were conducted to collect data for building an artificial neural network (ANN), using a single-cavity mold to produce a bowl-shaped product with a diameter of 99.90 mm and a height of 50.80 mm, as shown in Figure 2. Both the sides and the bottom of the product have a uniform thickness of 3.00 mm. The product was injection-molded using LG Chem’s polypropylene (PP), specifically LUPOL GP1007F, and the material properties provided by the manufacturer are shown in Table 3. For the injection-molding experiments, a 150-t injection molding machine (LGE II-150, LSMtron) was used.

### 2.2. Experimental Conditions

In the injection-molding experiments, six controllable variables were selected: melt temperature, mold temperature, injection speed, packing pressure, packing time, and cooling time. These six process variables are widely recognized as representative factors in injection-molding processes. They are commonly applied as process conditions in actual injection-molding analyses or experiments and are considered key process variables in various studies for optimizing injection-molding processes [3,4,6,8,9,11,12,13,15,16,17,18]. The melt temperature and mold temperature ranges for the injection-molding experiments were set from 200 °C to 240 °C at three levels, as shown in Table 4, based on the recommended conditions provided by the resin manufacturer (LG Chem, Seoul, Republic of Korea) and using material data from Autodesk Moldflow Insight 2023 (45.1.117), an injection-molding analysis software.

The injection speed and cooling time were derived through CAE analysis using Moldflow Insight 2023. For the injection speed, a level was determined through actual injection-molding experiments to ensure trouble-free product production. Then, the variation in injection time with respect to injection speed was interpreted up to this speed. Based on this, the range where injection speed and injection time exhibit a linear relationship was identified, and the operating levels for injection speed were set, as in Table 4. The cooling time was determined by analyzing the injection-molding process under extreme conditions to derive the minimum time required for the product to reach 100% solidification or eject temperature. An additional 10 s was then added to this time to determine the levels in Table 4. Packing conditions were determined through actual injection-molding experiments to ensure trouble-free product formation. Specifically, for packing time, the point at which the gate of the product solidifies, indicating the moment when no more material is injected, was identified. This defined the reference time, and the levels in Table 4 were derived accordingly.

**Figure 3:**
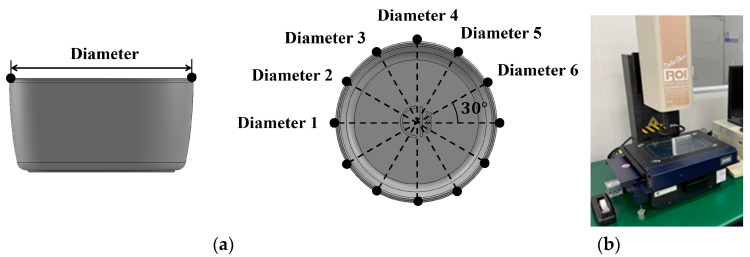
(**a**) Measurement points of bowl product; (**b**) measurement instrument (Datastar200, RAM OPTICAL INSTRUMENT, USA).

**Figure 4:**
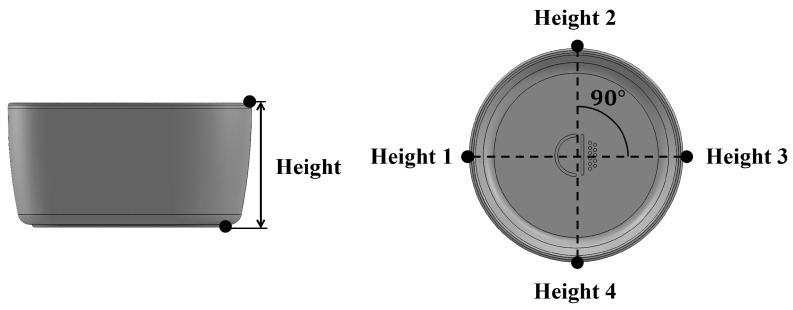
Measurement points of bowl product: height.

Table 5 lists 50 process conditions that reflect the factors and levels listed in Table 4. Based on the levels presented in Table 4, 27 process conditions were created by the orthogonal array of L27, and 23 process conditions were randomly generated within the corresponding range. Injection-molding experiments were conducted under the molding conditions shown in Table 5. The quality of the injection-molded products was evaluated by measuring their mass, diameter, and height. For the diameter measurement, the diameter at the entrance of the mold was recorded, with measurements taken at a total of six points and evaluated as an average, as shown in Figure 3. For the height measurement, the height was measured at four different points and the average value was calculated (Figure 4).

## 3. Neural Network Architectures and Implementation

### 3.1. Injection Molding

**Figure 5:**
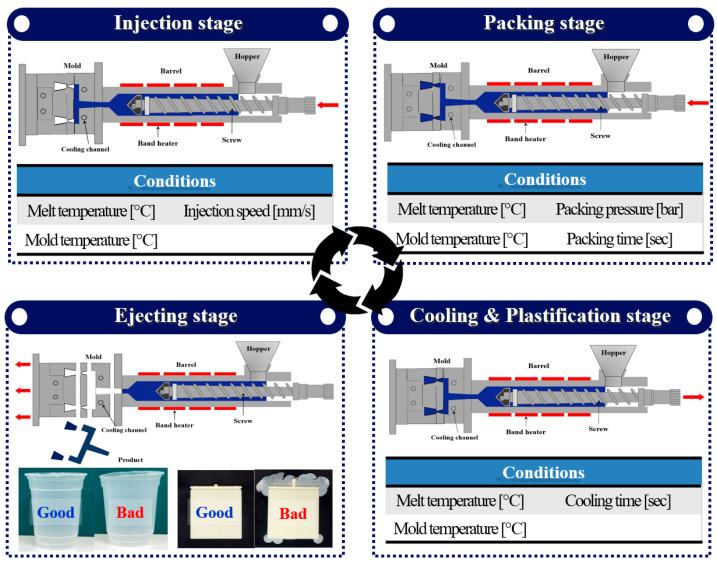
Injection-molding process stages and key process variables.

Injection-molding processes are broadly divided into four stages: injection, packing, cooling and plastification, and ejection. Figure 5 illustrates how these stages are configured and depicts the influence of various process variables on the injection-molding process. The injection stage is the phase in which the screw of the injection-molding machine is controlled by speed to initiate the filling of the material into the shape of the product within the mold. In this stage, there is a need to rapidly fill the material before it solidifies, and, typically, 98–99% of the entire product volume is filled. Therefore, in the injection stage, melt temperature, mold temperature, and injection speed act as critical process variables. The packing stage is a phase in which the volume of the unfilled product from the injection stage is filled by controlling the pressure. This stage is aimed at compensating for issues such as shrinkage in injection-molded products. In this stage, packing pressure and packing time influence the process, and additionally, factors such as melt temperature and mold temperature, which can affect the magnitude of pressure, also play a role. The outcome parameters from the preceding process stage (injection stage) also have an impact. The cooling stage is the phase that accounts for the majority of the cycle time in the injection-molding process, where the temperature of the fully formed product inside the mold is cooled to a temperature without any deformation. Therefore, the initially set melt temperature, mold temperature, and cooling time impact the process. It is also influenced by the outcome parameters of the injection and packing stages. The plastification stage occurs concurrently with the cooling stage, involving the preparation of the material for the next product production within the barrel of the injection-molding machine. This stage can be considered a kind of process preparation step, where the material is pre-melted at the set melt temperature and mold temperature. In this study, we considered three stages: injection, packing, and cooling. Additionally, the ejection stage involves separating the molded product from the mold upon completion and transferring it.

### 3.2. Neural Network Archtectures

**Figure 6:**
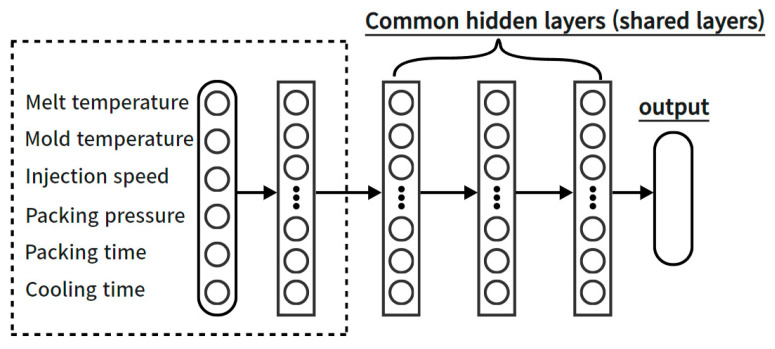
Network #1: structure in which the input parameters are connected into a single layer.

**Figure 7:**
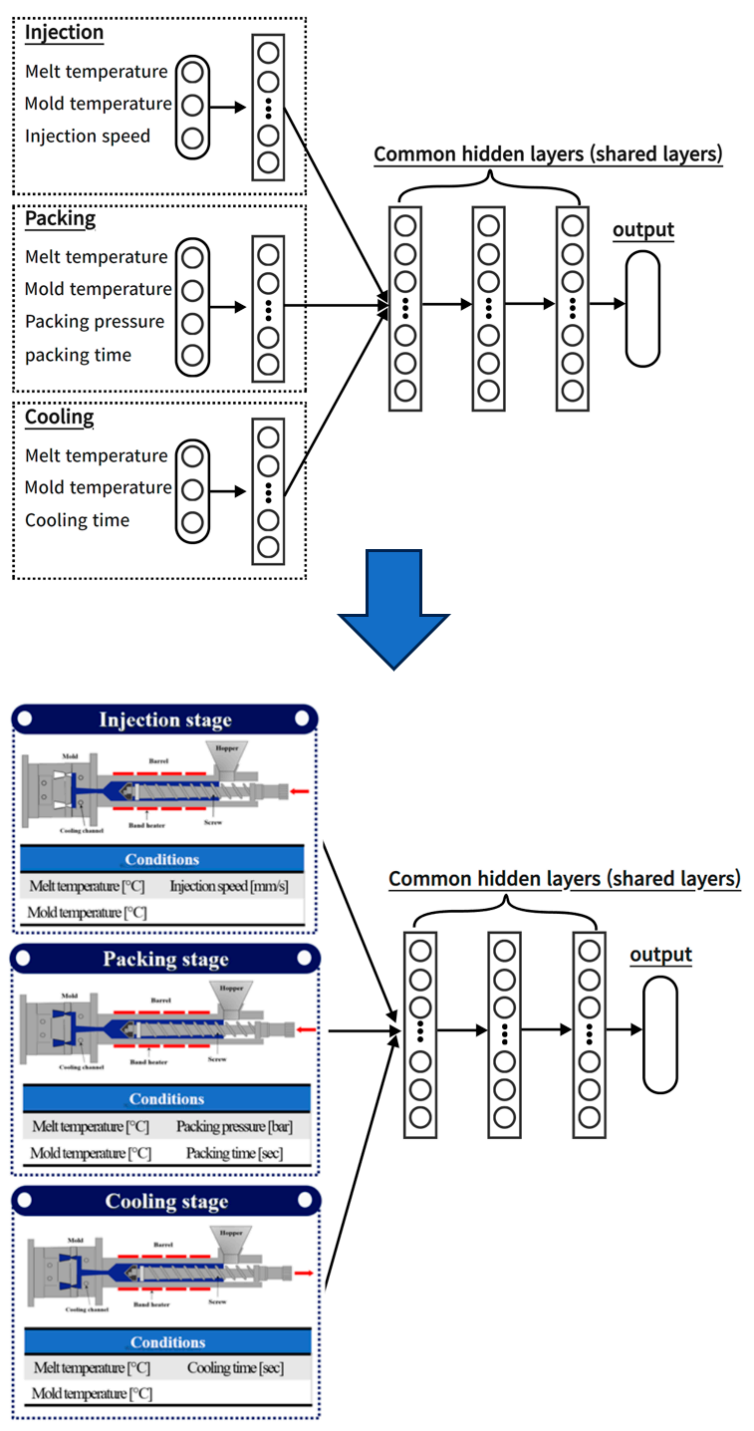
Network #2: structure where input parameters are simultaneously applied through layers that are differentiated according to the injection-molding process stages.

In this paper, three architectures were investigated and compared for predicting the quality of injection-molded products. One is a typical artificial neural network (ANN), similar to Network #1 in Figure 6, while the other follows a structure where input parameters are simultaneously applied through layers that are differentiated according to the injection-molding process stages, as seen in Network #2 in Figure 7. The structure of Network #2 in Figure 7 is grouped according to injection-molding processes based on input parameters; however, it is a non-interacting structure. In other words, the outcomes of the injection stage do not influence the packing stage, and the results of the packing stage do not affect the cooling stage. While input parameters are grouped by process, the sequence of the processes is not considered.

**Figure 8:**
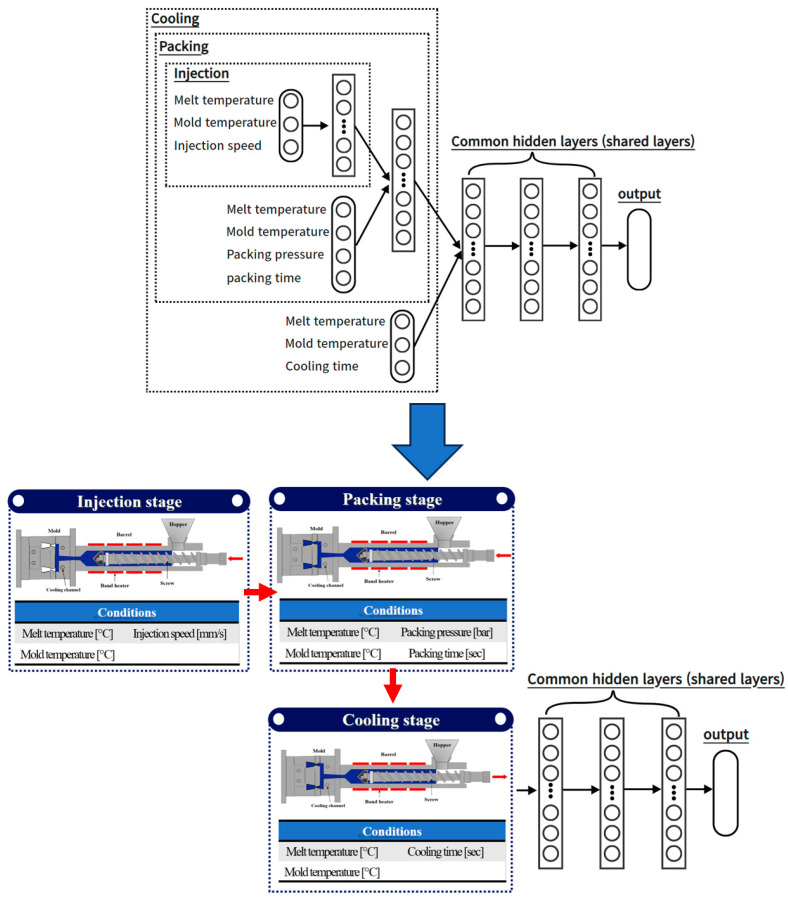
Network #3: structure where input parameters are differentiated according to the injection-molding process stages and described through continuous sequence layers.

The remaining architecture adopts a structure where input parameters are differentiated according to the injection-molding process stages and described through continuous sequence layers, similar to Network #3 in Figure 8. In this structure, input parameters are grouped by process, and the process sequence is also considered to allow interaction between the preceding and succeeding stages. Six different process conditions were used as input parameters, including melt temperature, mold temperature, injection speed, packing pressure, packing time, and cooling time. In networks #2 and #3, the input parameters are grouped into injection, packing, and cooling stages according to the structure and characteristics of the process steps to reflect the interactive influence between the processes from a macro perspective.

The melt temperature and mold temperature were arranged to affect all stages, and the injection speed was assigned to the injection group, the packing pressure and time to the packing group, and the cooling time to the cooling group and applied to the ANN. To facilitate a comprehensive comparison between the three architectures, a single-output model was built that separately predicted mass, diameter, and height as output parameters. Also, to allow for a straightforward comparison based on the structural differences of the three architectures, a consistent set of shared layers was maintained with a fixed number of three, while varying the structure of the input parameters and the hidden layer structures that receive them. However, only the number and structure of the hidden layers remained fixed, and the remaining hyperparameters were determined by an exploration process to find values suitable for the architecture.

### 3.3. Data Processing

From the total dataset of 50 process conditions in Table 5, 38 datasets were assigned as the training dataset, 6 datasets were assigned as the validation dataset, and the remaining 6 datasets were assigned as the test dataset to evaluate the performance of the model. The datasets were subjected to min–max normalization to standardize the magnitudes and differences in values among the parameters, ensuring that the influence based on the parameter scale was consistent. For the min–max normalization, Equation (1) was used, and the range was normalized to 0.1–0.9 to prevent saturation caused by data leakage.

(1) $${x^{\prime }}_{i}=\left(0.9-0.1\right)\times \left\{\frac{\left(x_{i}-\mathrm{Min}.\;\;X\right)}{\left(\mathrm{Max}.\;\;X-\mathrm{Min}.\;\;X\right)}\right\}+0.1,\;{x^{\prime }}_{i}\in X$$

### 3.4. The Search for Optimal Hyperparameters

During the process of training a machine learning model using ANNs, the parameters that users need to set are referred to as hyperparameters. Since the efficiency and performance of ANNs are determined by the initial values of these hyperparameters, it is important to configure suitable hyperparameters that align with the nature of the data and the objectives of the model. Therefore, the hyperband technique was used to determine appropriate hyperparameters, as shown in Table 6. The hyperband technique is widely used because it offers shorter optimization times and better performance results compared to conventional methods such as grid search, random search, and Bayesian search [22]. The number of hidden layers is also a critical hyperparameter that requires exploration. However, in this study, to facilitate the comparison of Networks #1, #2, and #3, the number and structures of the layers shown in Figure 6, Figure 7 and Figure 8 were used. Furthermore, various tools were applied to prevent underfitting and overfitting in the artificial neural network. The tools applied to the structure of the artificial neural network included Batch Normalization, Dropout, Weight Regularization (L2), Limitation of the number of neurons and layers to avoid complexity, and Early Stopping. To maintain the mean and covariance of the neural network, batch normalization was applied to each hidden layer. Additionally, ‘dropout’ was employed to prevent overfitting caused by excessively training specific variables. The dropout rate, considered a hyperparameter, was explored within the range of 0.0 to 0.4, tailored to the model and data. Values exceeding 0.5 could potentially degrade the model’s accuracy, hence we restricted the range to 0.4. Furthermore, L2 regularization, a form of weight regularization, was applied to prevent certain weight values from becoming too large and dependent on specific features, allowing the model to reflect general characteristics of the data. To limit the unrestricted increase in the complexity of the artificial neural network, the maximum number of neurons per layer was set to twice the sum of the input and output parameter counts. The minimum number of neurons that could be allocated was constrained by the output parameter count. Moreover, the total number of neurons placed throughout the entire artificial neural network was restricted within a predefined range to allow for convergence from input parameters to output parameters. This constraint was imposed by considering the number of neurons in the previous layer as a limiting range for the next layer, permitting a square or conical structure. This approach aimed to prevent excessive complexity in the model. Also, to compare the generalized neural network structure with the newly proposed structure, the study limited the shared hidden layer count to three, serving as a measure to prevent the model from becoming excessively complex. Typically, having more than four hidden layers in a neural network can lead to optimization issues, making it one of the factors contributing to the model’s excessive complexity. Finally, to prevent the neural network model from overfitting to the training dataset, the early stopping technique was applied. The endpoint of training was set based on a compromise value of the loss function between the training and validation datasets, preventing the model from overfitting to the training data.

In addition, the root mean square errors (RMSEs) from Equation (2) were used as a metric to evaluate the performance during the training process of the ANNs. In Equation (2), N represents the number of data used for evaluation, where $y_{i}$ corresponds to the measured values and $\hat{y_{i}}$ represents the predictions made by the ANN.

(2) $$\mathrm{RMSE}=\sqrt{\frac{1}{N}\sum_{i}^{N}{\left(y_{i}-\hat{y_{i}}\right)}^{2}}$$

## 4. Results

### 4.1. Relationship between Process Conditions and Injection-Molded Quality: Experiment Dataset

Table 7 shows the main effects between six process conditions and the mass of injection-molded products using the datasets generated by L27 orthogonal array design in Experiment #1–27 among the 50 datasets in Table 5. Table 8 indicates the contribution analysis of six process conditions to the mass of injection-molded products over the entire set of 50 datasets. Interactions were analyzed up to the second order. Examining the results in Table 7 and Table 8, it can be observed that the most significant factor influencing mass is the packing time. Following closely is the melt temperature as the next influential factor. Generally, the packing stage is known to have a close relationship with the mass of the molded product, as it involves controlling pressure to force additional material into the mold. Specifically, in this study, the mold used employs a direct hot runner, resulting in a delayed solidification time at the gate section. Therefore, in this study, the maximum packing time for feasible product production was applied, yielding results indicating that packing time is a significant contributor to mass. Subsequently, melt temperature, as a factor associated with the solidification time of the product or gate, can influence the mass of the molded product, as higher values are correlated with delayed solidification.

Table 9 shows the main effects of six process variables on the diameter of the molded product, while Table 10 illustrates the contribution analysis of these six process variables to the diameter. Analyzing the results from Table 9 and Table 10, it is evident that, similar to the mass, the most significant influence on diameter is attributed to the packing time. This is because, generally, the impact of the packing time on the diameter is substantial, as the product is typically forcibly charged through pressure control, compensating for factors such as shrinkage. Additionally, this substantial contribution is attributed to the prolonged maximum packing time applied in this study to produce the molded product. The remaining factors, such as mold temperature and packing pressure, also exhibit a relatively high influence on diameter compared to other factors. This can be attributed to product compensation and delayed solidification.

Table 11 and Table 12 present the main effects and contributions to the height of the injection-molded product. For the height of the injection-molded product, it is observed that both packing pressure and packing time exert significant influence. This is due to the practice of forcibly adding material through pressure control after the injection stage, which ultimately has a substantial impact on the dimensions of the product, similar to mass and diameter. In contrast, for the height of the molded product, the effects of interactions are relatively negligible compared to the main effect factors.

### 4.2. Comparison of the Single-Output Models with Mass as the Output Parameter

Table A1, Table A2 and Table A3 (Appendix A) show the results of hyperparameter exploration for Networks #1, #2, and #3, where mass is the output variable. While hyperparameter exploration was performed, it is important to note that the hidden layer structure was kept constant to facilitate an intuitive comparison between the three artificial neural network (ANN) architectures. The prediction results for the test data not used to train with the neural networks in Table A1, Table A2 and Table A3 (Appendix A) are shown in Table 13. Performance was compared by calculating the root mean square errors (RMSEs) value of the measured value and the predicted value of the neural network for the normalized test data.

The test data consists of the datasets (Exp. #28, 30, 31, 32, 36 and 45) from Table 5. As shown in Table 13, the best performance was achieved by Network #3, which passes the input parameters as a continuous sequence of injection-molding process steps in an ANN structure to predict mass. In the case of Network #2, it also showed a performance level comparable to the RMSE results for mass prediction achieved by Network #3.

**Figure 9:**
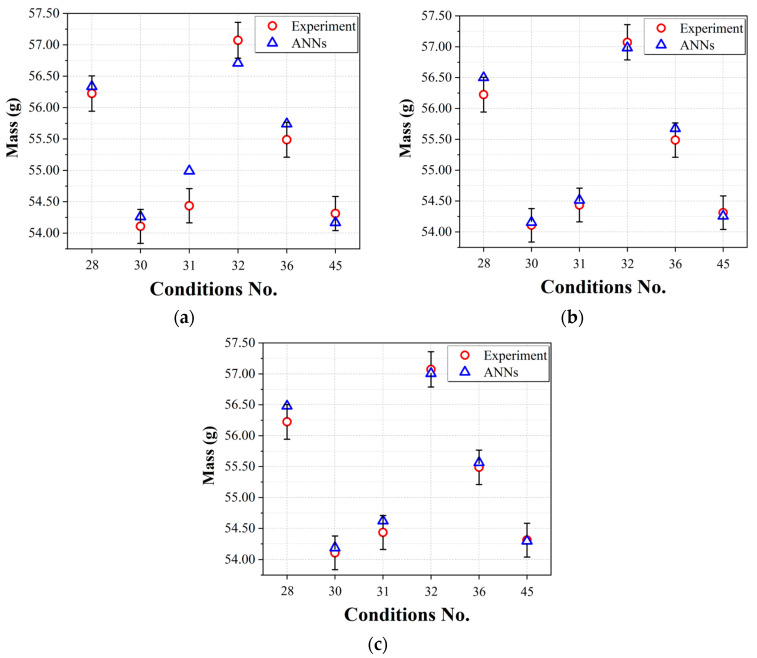
Mass prediction performances of the prediction models using test data: (**a**) Network #1; (**b**) Network #2; (**c**) Network #3.

Figure 9 shows the predicted mass results of three network architectures obtained from the test data and experimental results, with error bars calculated by the mass quality standard for general PP. A figure of ± 0.5% [18] was applied as the standard error of the mass for the PP molded product. According to the results in Figure 9, although the hyperparameter structures investigated in this study are not the optimal ones, it is evident that the predictions of Networks #2 and #3 meet the mass criteria, and an improvement can be observed compared to the results of Network #1. Based on these results, it can be confirmed that an architecture that incorporates the structure and characteristics of the injection-molding process outperforms typical ANNs in predicting the mass of the products used in this study.

### 4.3. Comparison of the Single-Output Models with Diameter as the Output Parameter

The hyperparameter values for the three network architectures used to predict the diameter of the injection-molded products in this study are shown in Table A4, Table A5 and Table A6 (Appendix A). In Table 14, as in Section 4.2, the diameter prediction results of each network architecture on the normalized test data are presented in terms of RMSEs.

**Figure 10:**
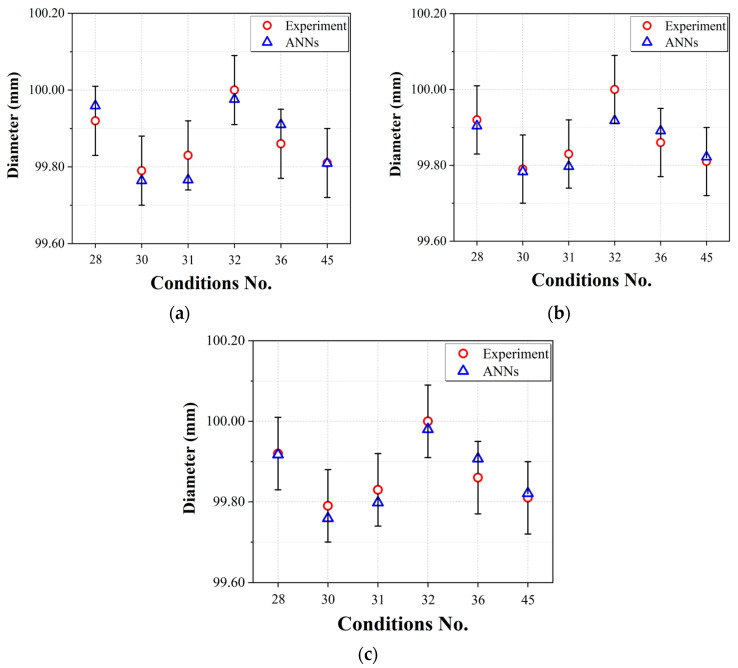
Diameter prediction performances of the prediction models using test data: (**a**) Network #1; (**b**) Network #2; (**c**) Network #3.

In the context of predicting the diameter of a product using ANNs, Network #1 showed the highest RMSE value, while Network #3 had the most favorable RMSE value. Network #2 had an RMSE value that was better than that of Network #1, but by a relatively small margin that warrants consideration at a comparable level. However, Network #3 clearly had a significantly lower RMSE than the other network architectures, confirming its superior performance in predicting the diameter. This can also be seen in the graph of the prediction results versus the actual product diameter and error bars calculated by applying ISO 20457:2018 [24] (Plastics molded parts—Tolerances and acceptance conditions); the dimensional quality standard for injection-molded products, shown in Figure 10. The calculated standard error equivalent to ISO 20457:2018 of the injection-molded product used in this study was ±0.09 mm. Network #1, a typical ANN, and Networks #2 and #3, which incorporate process steps into the connections between input parameters and hidden layers, all meet the length quality criteria. In particular, Network #3 produces predictions that are closer to the actual measurements. Based on these results, it can be seen that, even in the case of predicting the diameter of a bowl’s molded part, the ANN architecture that reflects the injection-molding process steps performs well and produces better results than the typical ANN (Network #1).

### 4.4. Comparison of the Single-Output Models with Height as the Output Parameter

**Figure 11:**
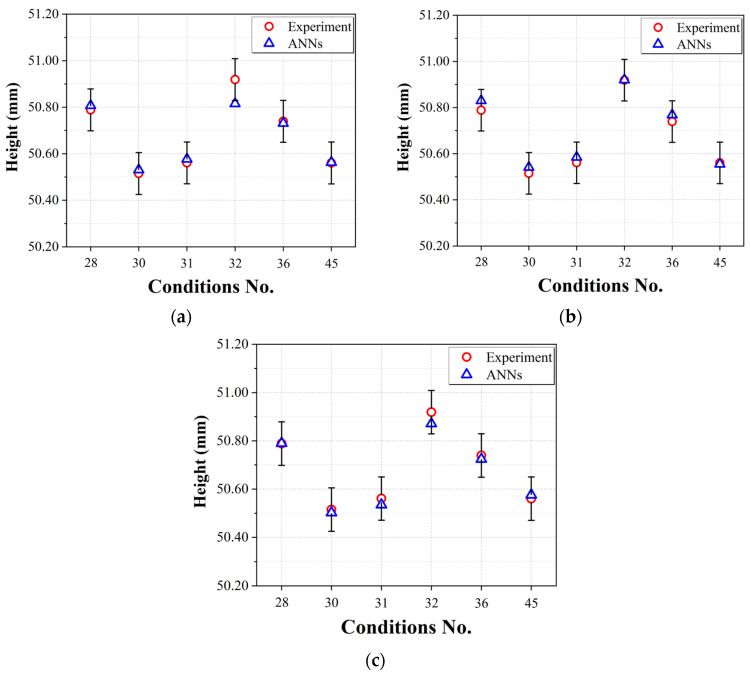
Height prediction performances of the prediction models using test data: (**a**) Network #1; (**b**) Network #2; (**c**) Network #3.

The results of hyperparameter exploration for ANN architectures with the height of the bowl product as the output parameter are presented in Table A7, Table A8 and Table A9 (Appendix A). The results of evaluating the performance of ANNs, Network #1, Network #2 and Network #3, with the applied selected hyperparameter values applied using test data are presented in Table 15. Compared to Network #1, which is a typical ANN structure, Network #2, 3 both showed better RMSE results by more than about twice. Figure 11 shows the actual height measurement data and error bars for the bowl product and compares the predictions from Networks #1, 2, and 3. The error bars were calculated by applying ISO 20457:2018 (Plastics molded parts—Tolerances and acceptance conditions), the dimensional quality standard for injection molded products, as was done for diameters in Section 4.3. Figure 10 shows that the neural network models from all three architectures predict the heights of the bowl products within the quality standards. Figure 11 also shows that Networks #2 and 3, which include the process steps, are very close to the actual measured data. Based on these results, it can be seen that an ANN structure (Network #2, 3) that reflects the process steps may be more suitable than the typical architecture (Network #1) for predicting the height of a molded part.

## 5. Conclusions

In this study, an artificial neural network (ANN) was built to predict the correlation between process conditions and product quality in injection molding. In order to evaluate the performance of architectures that incorporate process stages into the connection between input parameters and the hidden layer, injection-molding experiments were conducted on dish products and data were collected.

**Figure 12:**
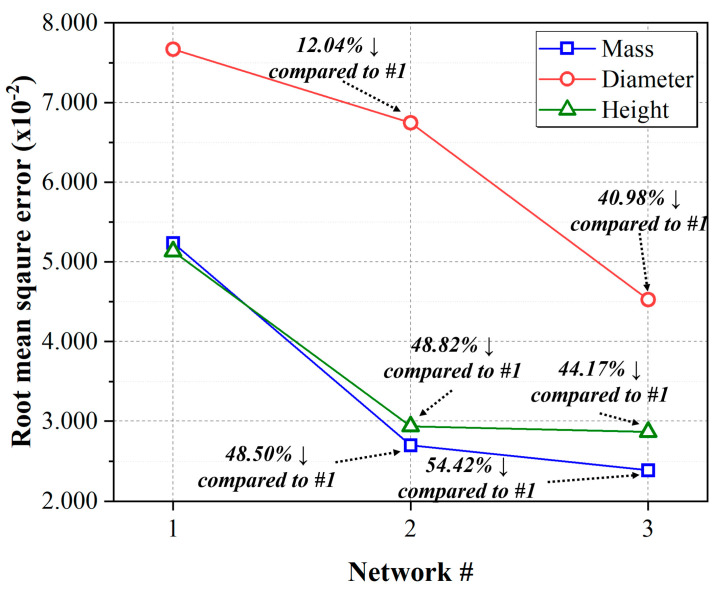
Performance improvement comparison relative to Network #1 (conventional).

Based on the collected dataset, three different ANN networks with different architectures were constructed. One follows the conventional ANN structure, where all input parameters are directly connected to the hidden layer. The other two ANNs apply the process stages and characteristics of injection molding to the input parameters. One groups input parameters based on the injection stage, packing stage, and cooling stage, and simultaneously feeds them into the hidden layers as a multi-task architecture. The other groups input parameters by process stage and configures the network to feed input parameters in a continuous sequence as the process stages progress. In the case of Networks #2 and #3, which applied the process steps, both showed relatively superior performance in predicting product quality in all scenarios compared to the typical ANN, Network #1. In particular, the architecture of Network #3, which transmits input parameters in a continuous sequence based on the process order, showed outstanding performance in predicting product mass, diameter, and length. Compared to the root mean square error (RMSE) value of Network #1, a typical artificial neural network, the RMSE of Network #3 for mass improved by approximately 54.42%, while diameter and height showed performance improvements of 40.98% and 44.17%, respectively, in terms of RMSE-based evaluations. For Network #2, there is also an improvement in performance, although not as significant as in the case of Network #3. The RMSE of Network #2 for mass, diameter, and height showed a reduction of 48.50%, 12.04%, and 42.82%, respectively, when compared to the RMSE of Network #1. In conclusion, this study showed that ANN architectures incorporating the injection-molding process steps as input parameters showed an average prediction performance improvement of approximately 46.52% for Network #3 and 34.45% for Network #2 based on RMSE performance. Figure 12 shows a diagram depicting how the new network performance has improved compared to the existing artificial neural network.

Furthermore, when comparing these results with quality criteria for injection-molded products, it was observed that the conventional artificial neural network failed to meet the quality standards in the mass prediction for Exp. #31 and #32, as well as in the height prediction for Exp. #32. In contrast, Network #2 and Network #3 confirmed that for mass, diameter, and height predictions, all test data fell within the quality standards based on improvement RMSE. This suggests that applying an ANN with input parameters grouped by process stages, instead of a conventional ANN, in real injection-molding processes and industry can lead to enhanced production rates through predictions within quality standards. Therefore, these results suggest that incorporating the structural aspects of each stage of the injection-molding process into the neural network architecture may be a more suitable approach to data prediction using ANNs.

From the analysis of the specific dataset for the bowl product used in this study, it might be the better choice for predicting the mass and length to construct an ANN based on multi-task learning, which incorporates the process steps and characteristics of injection-molding into the connections between input parameters and hidden layers, rather than the typical ANN. The approach employed in this study’s results can be applied to service systems, akin to other research outcomes [13,23] with a systemic perspective. In a system aimed at deriving the initial optimal conditions for molding products of the target quality in an injection-molding process, this approach enhances prediction accuracy, thereby improving user convenience and productivity. Furthermore, the results of this study can be applied to a predictive-based process control system, where changes in process conditions and quality maintenance are conducted through quality predictions, rather than through step-by-step process control. This application can enhance accuracy and productivity, while reducing the time consumed by condition changes for quality improvement and other processes. As with this use case, the results of this study might be used as a useful reference for future research on applying ANNs to the injection-molding industry, such as predictive process control, deriving optimal conditions, and building a digital twin injection-molding platform based on them.

## Figures and Tables

**Table 1:** Previous research on MISO model applied to the injection-molding process.

Author	Input Parameters	Output Parameters	The Number of Hidden Layers	The Number of Neurons Per Hidden Layers
Ozcelik, B et al. [11]	5 (Mold Temp., Melt Temp., Packing pressure, Packing time, Cooling time)	1 (Warpage)	2 hidden layers	9 (1st)–9 (2nd)
Yin, F et al. [12]	5 (Mold Temp., Melt Temp., Packing pressure, Packing time, Cooling time)	1 (Warpage)	2 hidden layers	20 (1st)–20 (2nd)
Lee, C. H et al. [13]	9 (Overall volume, Cavity volume, Overall surface area, Cavity surface area, Filling time, Melt Temp., Mold Temp., Packing pressure, Packing time)	1 (Weight)	2 hidden layers	28 (1st)–28 (2nd)
Gim, J. et al. [14]	10 (Time and pressure value from sensor)	1 (Part weight)	1 hidden layer	8

**Table 2:** Previous research on MIMO applied to the injection-molding process.

Author	Input Parameters	Output Parameters	The Number of Hidden Layers	The Number of Neurons Per Hidden Layers
Abdul, R et al. [15]	3 (Injection speed, Holding time, Cooling time)	2 (Length shrinkage, Width shrinkage)	1 hidden layer	4 (1st)
Heinisch, J et al. [16]	6 (Mold Temp., Melt Temp., Injection time, Packing pressure, Packing time, Cooling time)	3 (Weight, Length, Width)	1 hidden layer	5 (1st)
Huang, Y. M. et al. [17]	5 (Injection speed, Packing time, Mold Temp., Melt Temp.)	3 (Injection pressure, Cooling time, Z shrinkage)	2 hidden layers	7 (1st)–3 (2nd)
Gim, J. et al. [14]	10 (Time and pressure value from sensor)	5 (Injection pressure, Cooling time, X, Y, Z shrinkage)	2 hidden layers	11 (1st)–7 (2nd)
Lee. J. H. et al. [18]	6 (Melt Temp., Mold Temp., Injection speed, Packing pressure, Packing time, Cooling time)	3 (Mass, Diameter, Height)	2 shared hidden layers, 1 specific-task hidden layer	6 (1st)-5 (2nd)-[4(mass), 3(diameter), 4(height)]

**Table 3:** Material properties of the polypropylene (LUPOL GP1007F, LG Chemical Co. Ltd., Seoul, Republic of Korea) [21].

Properties		Standard	Condition	Unit	Value
Physical	Specific gravity	ASTM D792	-	-	0.94
	Melt flow rate	ASTM D1238	230 °C,	g/10 min	13.0
			2.16 kg		
Mechanical	Tensile strength	ASTM D638	50 mm/min	kgf/cm^2^	290
	(3.2 mm)				
	Flexural strength	ASTM D790	10 mm/min	kgf/cm^2^	380
	(6.4 mm)				
Thermal	Heat deflection Temp. (6.4 mm)	ASTM D648	4.6 kg	°C	110

**Table 4:** Process conditions and levels for the injection-molding experiment.

Conditions	Level 1	Level 2	Level 3
Melt temperature (°C)	200	220	240
Mold temperature (°C)	40	50	60
Injection speed (mm/s)	40	70	100
Packing pressure (bar)	150	200	250
Packing time (s)	6.0	12.0	18.0
Cooling time (s)	38	48	58

**Table 5:** Injection-molding conditions generated by orthogonal array of L27 and random array. Reprinted/adapted with permission from Ref. [18]. 2022, Lee, J. H.; Yang, D. C.; Yoon, K. H.; Kim, J. S.

Exp. No.	Melt Temperature (°C)	Mold Temperature (°C)	Injection Speed (mm/s)	Packing Pressure (bar)	Packing Time (s)	Cooling Time (s)	Note
1	200	40	40.0	150	6.0	38	L27
2	200	40	40.0	150	12.0	48	L27
3	200	40	40.0	150	18.0	58	L27
4	200	50	70.0	200	6.0	38	L27
5	200	50	70.0	200	12.0	48	L27
6	200	50	70.0	200	18.0	58	L27
7	200	60	100.0	250	6.0	38	L27
9	200	60	100.0	250	18.0	58	L27
10	220	40	70.0	250	6.0	48	L27
11	220	40	70.0	250	12.0	58	L27
12	220	40	70.0	250	18.0	38	L27
13	220	50	100.0	150	6.0	48	L27
14	220	50	100.0	150	12.0	58	L27
15	220	50	100.0	150	18.0	38	L27
16	220	60	40.0	200	6.0	48	L27
17	220	60	40.0	200	12.0	58	L27
18	220	60	40.0	200	18.0	38	L27
19	240	40	100.0	200	6.0	58	L27
20	240	40	100.0	200	12.0	38	L27
21	240	40	100.0	200	18.0	48	L27
22	240	40	40.0	250	6.0	58	L27
23	240	50	40.0	250	12.0	38	L27
24	240	50	40.0	250	18.0	48	L27
25	240	60	70.0	150	6.0	58	L27
26	240	60	70.0	150	12.0	38	L27
27	240	60	70.0	150	18.0	48	L27
28	214	55	82.7	204	16.3	52	Random
29	204	44	43.4	202	13.9	41	Random
30	203	46	93.6	205	13.7	45	Random
31	202	54	83.4	213	6.6	48	Random
32	206	43	61.6	221	6.9	39	Random
33	212	44	53.3	240	17.0	52	Random
34	212	51	90.8	224	6.1	48	Random
35	200	52	50.0	215	17.6	39	Random
36	229	51	46.2	153	11.7	45	Random
37	228	49	53.2	217	12.3	58	Random
38	222	51	63.7	167	8.7	51	Random
39	219	50	41.4	156	16.3	52	Random
40	228	46	96.5	154	16.7	57	Random
41	228	46	62.5	191	10.9	46	Random
42	219	42	98.4	237	17.9	41	Random
43	220	43	55.8	241	14.8	44	Random
44	233	42	50.8	198	13.5	55	Random
45	238	53	41.6	221	17.2	40	Random
46	234	48	68.2	222	8.8	41	Random
47	233	44	84.9	171	6.7	55	Random
48	234	43	56.9	176	11.1	48	Random
49	239	49	41.2	234	8.6	52	Random
50	240	49	76.1	241	6.4	51	Random

**Table 6:** Ranges of hyperparameters for Networks.

Hyperparameters	Range	Note
Seed number	0–50	Step size was 1
Batch size	16, 32, 64,…	Increased in multiples of 2 until it could cover the number of learning data
Optimizer	Adams [23]	Fixed
Learning rate	0.0001–0.01 [23]	Step size was 0.0001
Beta 1	0.1–1.0 [23]	Step size was 0.1
Bata 2	0.9, 0.99, 0.999, 0.999 [23]	-
Number of neurons	From the number of output parameters to twice the sum of the number of output and input parameters.	Step size was 1
Initializer	He normal (hidden layer)	-
	Xavier normal (output layer)	
Activation function	Elu (hidden layer)	-
	Linear (output layer)	
Drop number	0.0–0.4	Step size was 0.1
Coefficient of L2 normalization	0.001, 0.01, 0.1	-

**Table 7:** Main effects of L27 orthogonal array (DOE) on dataset: mass.

Process Variable	Mean Value of Mass (g)			Rank
	Level 1	Level 2	Level 3	
Melt temperature	55.68	55.20	54.70	2
Mold temperature	55.44	55.22	54.91	4
Injection speed	55.31	55.20	55.06	5
Packing pressure	54.77	55.22	55.58	3
Packing time	53.74	55.29	56.55	1
Cooling time	55.13	55.19	55.25	6

**Table 8:** Contribution analysis of the entire 50 datasets: mass.

Process Variable	Description	Contribution for Mass (%)
Melt temperature	Main effect	10.61
Mold temperature	Main effect	3.19
Injection speed	Main effect	1.83
Packing pressure	Main effect	4.64
Packing time	Main effect	78.77
Cooling time	Main effect	0.21
Melt temperature × Mold temperature	2-way interaction	0.02
Melt temperature × Injection speed	2-way interaction	0.01
Melt temperature × Packing pressure	2-way interaction	0.00
Melt temperature × Packing time	2-way interaction	0.00
Melt temperature × Cooling time	2-way interaction	0.00
Mold temperature × Injection speed	2-way interaction	0.00
Mold temperature × Packing pressure	2-way interaction	0.00
Mold temperature × Packing time	2-way interaction	0.08
Mold temperature × Cooling time	2-way interaction	0.02
Injection speed × Packing pressure	2-way interaction	0.01
Injection speed × Packing time	2-way interaction	0.05
Injection speed × Cooling time	2-way interaction	0.01
Packing pressure × Packing time	2-way interaction	0.00
Packing pressure × Cooling time	2-way interaction	0.00
Packing time × Cooling time	2-way interaction	0.01

**Table 9:** Main effects of L27 orthogonal array (DOE) on dataset: diameter.

Process Variable	Mean Value of Diameter (mm)			Rank
	Level 1	Level 2	Level 3	
Melt temperature	99.86	99.86	88.96	6
Mold temperature	99.86	99.87	99.85	4
Injection speed	99.86	99.85	99.87	5
Packing pressure	99.84	99.86	99.89	2
Packing time	99.72	99.90	99.96	1
Cooling time	99.86	99.88	99.84	3

**Table 10:** Contribution analysis of the entire 50 datasets: diameter.

Process Variable	Description	Contribution for Diameter (%)
Melt temperature	Main effect	0.11
Mold temperature	Main effect	1.98
Injection speed	Main effect	0.66
Packing pressure	Main effect	1.88
Packing time	Main effect	76.19
Cooling time	Main effect	0.40
Melt temperature × Mold temperature	2-way interaction	0.62
Melt temperature × Injection speed	2-way interaction	2.30
Melt temperature × Packing pressure	2-way interaction	0.32
Melt temperature × Packing time	2-way interaction	1.83
Melt temperature × Cooling time	2-way interaction	1.30
Mold temperature × Injection speed	2-way interaction	0.03
Mold temperature × Packing pressure	2-way interaction	0.05
Mold temperature × Packing time	2-way interaction	0.42
Mold temperature × Cooling time	2-way interaction	1.71
Injection speed × Packing pressure	2-way interaction	0.50
Injection speed × Packing time	2-way interaction	0.00
Injection speed × Cooling time	2-way interaction	0.60
Packing pressure × Packing time	2-way interaction	0.37
Packing pressure × Cooling time	2-way interaction	0.11
Packing time × Cooling time	2-way interaction	0.01

**Table 11:** Main effects of L27 orthogonal array (DOE) on dataset: height.

Process Variable	Mean Value of Diameter (mm)			Rank
	Level 1	Level 2	Level 3	
Melt temperature	50.72	50.67	50.62	6
Mold temperature	50.70	50.68	50.62	4
Injection speed	50.67	50.67	50.66	5
Packing pressure	50.58	50.67	50.75	2
Packing time	50.47	50.69	50.84	1
Cooling time	50.66	50.68	50.66	3

**Table 12:** Contribution analysis of the entire 50 datasets: height.

Process Variable	Description	Contribution for Diameter (%)
Melt temperature	Main effect	5.73
Mold temperature	Main effect	4.45
Injection speed	Main effect	0.95
Packing pressure	Main effect	10.27
Packing time	Main effect	75.78
Cooling time	Main effect	0.06
Melt temperature × Mold temperature	2-way interaction	0.23
Melt temperature × Injection speed	2-way interaction	0.17
Melt temperature × Packing pressure	2-way interaction	0.03
Melt temperature × Packing time	2-way interaction	0.23
Melt temperature × Cooling time	2-way interaction	0.00
Mold temperature × Injection speed	2-way interaction	0.00
Mold temperature × Packing pressure	2-way interaction	0.03
Mold temperature × Packing time	2-way interaction	0.00
Mold temperature × Cooling time	2-way interaction	0.01
Injection speed × Packing pressure	2-way interaction	0.04
Injection speed × Packing time	2-way interaction	0.07
Injection speed × Cooling time	2-way interaction	0.01
Packing pressure × Packing time	2-way interaction	0.07
Packing pressure × Cooling time	2-way interaction	0.01
Packing time × Cooling time	2-way interaction	0.06

**Table 13:** Root mean square errors (RMSEs) of normalized mass test data for Network #1, 2, 3.

Predicted Parameter	Network		
	#1	#2	#3
Mass	$5.237\times 10^{-2}$	$2.697\times 10^{-2}$	$2.387\times 10^{-2}$

**Table 14:** Root mean square errors (RMSEs) of normalized diameter test data for Network #1, 2, 3.

Predicted Parameter	Network		
	#1	#2	#3
Diameter	$7.669\times 10^{-2}$	$6.746\times 10^{-2}$	$4.526\times 10^{-2}$

**Table 15:** Root mean square errors (RMSEs) of normalized height test data for Network #1, 2, 3.

Predicted Parameter	Network		
	#1	#2	#3
Height	$5.133\times 10^{-2}$	$2.935\times 10^{-2}$	$2.866\times 10^{-2}$

## Data Availability

Data are contained within the article.

## Appendix A

**Table A1:** Hyperparameters of Network #1 architecture with mass as the output parameter.

Hyperparameters	Value
Seed number	16
Batch size	16
Optimizer	Adams
Learning rate	0.0027
Beta 1	0.7
Beta 2	0.999
Number of hidden layers	4
Number of neurons	12-6-6-3
Initializer	He normal (hidden layers) Xavier normal (output layer)
Activation function	Elu
Drop number	0.0-0.0-0.1-0.3
Coefficient of L2 normalization	0.001

**Table A2:** Hyperparameters of Network #2 architecture with mass as the output parameter.

Hyperparameters	Value
Seed number	41
Batch size	16
Optimizer	Adams
Learning rate	0.0086
Beta 1	0.9
Beta 2	0.999
Number of hidden layers	1 (process layer for injection stage)
	1 (process layer for packing stage)
	1 (process layer for cooling stage)
	3 (common layers)
Number of neurons	7 (process layer for injection stage)
	7 (process layer for packing stage)
	4 (process layer for cooling stage)
	13-13-8 (common layers)
Initializer	He normal (hidden layers)
	Xavier normal (output layer)
Activation function	Elu
Drop number	0.0 (process layer for injection stage)
	0.3 (process layer for packing stage)
	0.0 (process layer for cooling stage)
	0.1-0.1-0.2 (common layers)
Coefficient of L2 normalization	0.001

**Table A3:** Hyperparameters of Network #3 architecture with mass as the output parameter.

Hyperparameters	Value
Seed number	9
Batch size	16
Optimizer	Adams
Learning rate	0.0076
Beta 1	0.2
Beta 2	0.9999
Number of hidden layers	1 (process layer for injection stage)
	1 (process layer for packing stage)
	3 (common layers)
Number of neurons	2 (process layer for injection stage)
	9 (process layer for packing stage)
	8-4-1 (common layers)
Initializer	He normal (hidden layers)
	Xavier normal (output layer)
Activation function	Elu
Drop number	0.2 (process layer for injection stage)
	0.0 (process layer for packing stage)
	0.3-0.1-0.0 (common layers)
Coefficient of L2 normalization	0.001

**Table A4:** Hyperparameters of Network #1 architecture with diameter as the output parameter.

Hyperparameters	Value
Seed number	4
Batch size	32
Optimizer	Adams
Learning rate	0.0029
Beta 1	0.3
Beta 2	0.99
Number of hidden layers	4
Number of neurons	5-5-4-2
Initializer	He normal (hidden layers)
	Xavier normal (output layer)
Activation function	Elu
Drop number	0.2-0.2-0.1-0.2
Coefficient of L2 normalization	0.001

**Table A5:** Hyperparameters of Network #2 architecture with diameter as the output parameter.

Hyperparameters	Value
Seed number	25
Batch size	16
Optimizer	Adams
Learning rate	0.0043
Beta 1	0.8
Beta 2	0.9
Number of hidden layers	1 (process layer for injection stage)
	1 (process layer for packing stage)
	1 (process layer for cooling stage)
	3 (common layers)
Number of neurons	8 (process layer for injection stage)
	9 (process layer for packing stage)
	4 (process layer for cooling stage)
	21-14-14 (common layers)
Initializer	He normal (hidden layers)
	Xavier normal (output layer)
Activation function	Elu
Drop number	0.0 (process layer for injection stage)
	0.3 (process layer for packing stage)
	0.1 (process layer for cooling stage)
	0.2-0.1-0.4 (common layers)
Coefficient of L2 normalization	0.01

**Table A6:** Hyperparameters of Network #3 architecture with diameter as the output parameter.

Hyperparameters	Value
Seed number	44
Batch size	16
Optimizer	Adams
Learning rate	0.006
Beta 1	0.2
Beta 2	0.99
Number of hidden layers	1 (process layer for injection stage)
	1 (process layer for packing stage)
	3 (common layers)
Number of neurons	6 (process layer for injection stage)
	9 (process layer for packing stage)
	15-10-1 (common layers)
Initializer	He normal (hidden layers)
	Xavier normal (output layer)
Activation function	Elu
Drop number	0.3 (process layer for injection stage)
	0.0 (process layer for packing stage)
	0.1-0.3-0.2 (common layers)
Coefficient of L2 normalization	0.001

**Table A7:** Hyperparameters of Network #1 architecture with height as the output parameter.

Hyperparameters	Value
Seed number	30
Batch size	16
Optimizer	Adams
Learning rate	0.0019
Beta 1	0.2
Beta 2	0.9999
Number of hidden layers	4
Number of neurons	8-3-2-2
Initializer	He normal (hidden layers)
	Xavier normal (output layer)
Activation function	Elu
Drop number	0.3-0.1-0.1-0.0
Coefficient of L2 normalization	0.01

**Table A8:** Hyperparameters of Network #2 architecture with height as the output parameter.

Hyperparameters	Value
Seed number	35
Batch size	16
Optimizer	Adams
Learning rate	0.0089
Beta 1	0.2
Beta 2	0.9999
Number of hidden layers	1 (process layer for injection stage)
	1 (process layer for packing stage)
	1 (process layer for cooling stage)
	3 (common layers)
Number of neurons	8 (process layer for injection stage)
	6 (process layer for packing stage)
	3 (process layer for cooling stage)
	17-12-5 (common layers)
Initializer	He normal (hidden layers)
	Xavier normal (output layer)
Activation function	Elu
Drop number	0.1 (process layer for injection stage)
	0.2 (process layer for packing stage)
	0.0 (process layer for cooling stage)
	0.4-0.0-0.3 (common layers)
Coefficient of L2 normalization	0.01

**Table A9:** Hyperparameters of Network #3 architecture with height as the output parameter.

Hyperparameters	Value
Seed number	48
Batch size	16
Optimizer	Adams
Learning rate	0.0067
Beta 1	0.3
Beta 2	0.9999
Number of hidden layers	1 (process layer for injection stage)
	1 (process layer for packing stage)
	3 (common layers)
Number of neurons	1 (process layer for injection stage)
	9 (process layer for packing stage)
	6-6-3 (common layers)
Initializer	He normal (hidden layers)
	Xavier normal (output layer)
Activation function	Elu
Drop number	0.2 (process layer for injection stage)
	0.4 (process layer for packing stage)
	0.0-0.3-0.0 (common layers)
Coefficient of L2 normalization	0.001
